# Adipose Tissue and Renal Carcinoma: A Protumor Metabolic and Endocrine Alliance

**DOI:** 10.3390/ijms27031528

**Published:** 2026-02-04

**Authors:** Matías Ferrando, Daiana Lorena Moya Morales, Leonardo Rafael Romeo, Mauro Agustín Carrillo, Rocío Yasmin Cano, Silvina Esther Gómez, Constanza Matilde López-Fontana, Rubén Walter Carón, Flavia Alejandra Bruna, Virginia Pistone-Creydt

**Affiliations:** 1 Instituto de Medicina y Biología Experimental de Cuyo (IMBECU), Centro Científico y Tecnológico Mendoza, Consejo Nacional de Investigaciones Científicas y Técnicas (CONICET), Universidad Nacional de Cuyo, Mendoza M5500, Argentina; matiasferrando13@gmail.com (M.F.); daaimoya@gmail.com (D.L.M.M.); leonardo.romeo1@gmail.com (L.R.R.); urologia.carrillo@gmail.com (M.A.C.); rcano@mendoza-conicet.gob.ar (R.Y.C.); sgomez@mendoza-conicet.gob.ar (S.E.G.); clopezfontana@mendoza-conicet.gob.ar (C.M.L.-F.); rcaron@mendoza-conicet.gob.ar (R.W.C.); flabruna@gmail.com (F.A.B.); 2 Urocuyo, Mendoza M5500, Argentina; 3 Centro de Investigaciones Odontológicas, Facultad de Odontología, Universidad Nacional de Cuyo, Mendoza M5500, Argentina; 4 Instituto de Fisiología, Facultad de Ciencias Médicas, Universidad Nacional de Cuyo, Mendoza M5500, Argentina

**Keywords:** renal carcinoma, tumor microenvironment, adipocyte–tumor crosstalk, metabolic reprogramming, adipose tissue browning

## Abstract

Cancer is a multifactorial disease influenced not only by genetic and epigenetic alterations but also by interactions with the surrounding microenvironment. Among the hallmarks of cancer, metabolic reprogramming enables tumor cells to adapt and survive under adverse conditions. These metabolic alterations also induce changes in stromal cells. In clear cell renal cell carcinoma (ccRCC), adipocytes are among the most abundant stromal components. We have previously shown that ccRCC progression depends on the bidirectional crosstalk between tumor epithelial cells and neighboring adipocytes. Here, we investigated the effects of ccRCC on naïve human adipose tissue (hRAN). Human retroperitoneal adipose tissue fragments from two distinct donors (*n* = 2) were incubated with conditioned media (CMs) derived from ccRCC tumors (T-CM) or renal epithelial cells (Tc-CM). We analyzed the expression of adipocytokines, differentiation and browning markers, metabolic parameters, and steroid hormone receptor profiles. The exposure of hRAN to T-CM or Tc-CM led to significant alterations in the expression of adiponectin and leptin, as well as markers associated with differentiation and browning, including PLIN1, HSL, PGC1α, PPARγ, and UCP1. Adipocytes from treated hRAN were smaller than those from controls, suggesting dedifferentiation. Moreover, expression of FABP4 and MCT1 was significantly increased in explants treated with T-CM compared to control media. Conditioned media from these treated hRAN samples showed elevated lactate secretion, indicating enhanced lactatogenesis. Given the role of sex hormones in metabolic regulation, we examined the expression of estrogen (ER), androgen (AR), and progesterone (PR) receptors. While AR and PR levels remained unchanged, both ERα and ERβ were significantly upregulated after T-CM treatment. Metabolic reprogramming in renal tumors induces profound adaptive changes in adjacent adipose tissue. The dedifferentiation and browning of adipocytes, altered adipocytokine expression, and increased lactate production observed in hRAN reflect the metabolic stress imposed by the tumor environment. Here, we provide evidence, using an ex vivo model, of a dynamic partnership between human adipose tissue and ccRCC tumors.

## 1. Introduction

Cancer is a complex, multifactorial disease with a global impact. Among the cancer hallmarks [1], metabolic reprogramming allows tumor cells to survive in hostile environments [2]. Cancer cells need to adjust their metabolic programs to adapt to a constantly changing situation. Otto Warburg postulated that tumor origins lie in a change in cellular metabolism, primarily a decrease in oxidative phosphorylation (dependent on mitochondrial activity) and an increase in glycolysis, even in the presence of oxygen [3]. Since glycolysis is less efficient than oxidative phosphorylation in terms of bioenergetics, glycolysis can be accelerated up to 300-fold in tumor cells, resulting in high lactate production [4,5,6,7]. Lactate can be metabolized by the cell itself or exported to the extracellular matrix (ECM) by monocarboxylate transporters (MCTs) [8]. Lactic acid deposition in the ECM causes an acidification of the environment that allows tumor expansion and metastasis, reduces the immune response, and stimulates vasculogenesis [5,7,9,10,11,12,13]. This tumor metabolic change in turn generates a metabolic reprogramming of stromal cells, a process known as the “reverse Warburg effect”. According to this model, cancer cells induce glycolysis in adjacent cells [14,15], and metabolic changes in stromal cells may contribute to cancer pathogenesis [16].

Among the different cell types that comprise the tumor microenvironment heterogeneity in clear cell renal cell carcinoma (ccRCC), adipocytes are one of the most abundant. We recently demonstrated that tumor progression in ccRCC depends on the bidirectional crosstalk between tumor epithelial cells and neighboring stromal cells [17]. Specifically, human renal adipose tissue from ccRCC patients (hRAT) regulates the behavior of tumor and nontumor human renal epithelial cells differently than human renal adipose tissue from healthy individuals (hRAN). We observed that hRAT expresses significantly higher amounts of leptin, leptin receptor (ObR), and versican, as well as significantly lower amounts of perilipin, adiponectin, and adiponectin receptor (AdipoR1), compared to hRAN. However, we could not conclude that the presence of the renal tumor is responsible for this change in the secretion of adipocytokines and their receptors in perirenal adipose tissue (AT). We also demonstrate that hRAT-conditioned media (CMs) increase the metastatic capacity of tumor and nontumor human renal epithelial cells [18].

When adipocytes have an altered phenotype compared to normal adipocytes, they are called cancer-associated adipocytes (CAAs), and their differentiation appears to be an important aspect of tumorigenesis [19,20]. This reprogramming is regulated, at least in part, by soluble tumor-derived factors, such as proinflammatory cytokines (IL-6, TNF-α) and exosomal miRNAs, which trigger the dedifferentiation of mature adipocytes [21]. During this process, adipocytes lose differentiation markers such as PPARγ, exhibit reduced lipid droplet size due to accelerated lipolysis, and acquire a morphology similar to that of fibroblasts [22]. Furthermore, this microenvironment modified by the presence of the tumor not only affects mature adipocytes but also regulates metabolic changes in adipose-derived stem cells (ADCs), thereby promoting tumor development [6]. We have shown that adipocytes from hRAT patients exhibit a much smaller size compared to hRAN adipocytes and also undergo a browning process, supported by the significantly higher expression of a plethora of browning markers such as UCP1, TBX1, PPARγ, PCG1α, c/EBPα LAP, and c/EBPα LIP in hRAT vs. hRAN [18]. However, we do not yet know whether the presence of the renal tumor is responsible for the greater browning observed in hRAT.

In addition to homeostatic and metabolic functions, AT has endocrine and immunomodulatory functions and represents an extragonadal site of production of steroid hormones such as estrogens, androgens, and progesterone [23]. These types of steroids are modulators of the differentiation and proliferation of preadipocytes, of lipogenesis and lipolysis in mature adipocytes, and of the production of adipocytokines [24,25]. Steroid hormones and their receptors play important roles in the development of pathologies such as cancer [26]. The most common molecular subtype of RCC is clear cell carcinoma. It has been reported that ccRCC presents an accumulation of lipids in the cytoplasm, such as cholesterol and cholesterol esters [27]. Given that steroid hormones are derived from cholesterol [28], and that the incidence of the disease varies with sex [29], it would be expected that renal tumors would be hormone-sensitive and have steroidogenic capacity. Tumor steroidogenesis could impact the biology of the adjacent AT, in addition to its autocrine role in hormone production [30,31]. Lee et al., 2017 [32], have demonstrated intratumoral steroidogenesis, with in vivo and in vitro models of RCC, by evaluating androgen biosynthesis. We hypothesize that the perirenal adipose tissue modifies its characteristics in response to the adjacent tumor and that these adaptations could create a permissive microenvironment that drives tumor progression.

## 2. Results

### 2.1. The Expression of Browning Markers in hRAN Explants Incubated with Tc-CM Differed from That of NTc-CM and/or Ctrl-CM

**Figure 1:**
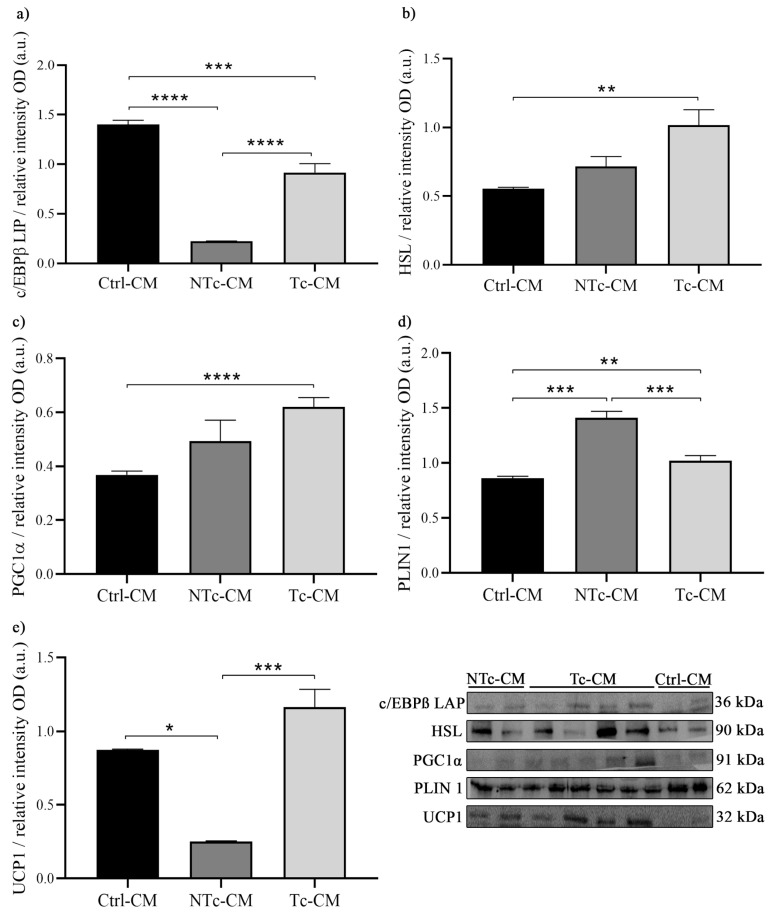
Expression of (**a**) c/EBPβ LAP, (**b**) HSL, (**c**) PGC1α, (**d**) PLIN1, and (**e**) UCP1 in hRAN incubated for 48 h with NTc- (*n* = 2), Tc- (*n* = 4), and Ctrl-CM (*n* = 2) by WB. The assay was performed in three technical replicates. Vertical bars represent the geometric mean of the data set, and horizontal bars represent SEM. Data were analyzed by Kruskal–Wallis or Brown–Forsythe tests, as appropriate. The expression of each of the evaluated proteins was relative to the total amount of protein by Ponceau staining. The values of *n* refer to biological replicates. * *p* < 0.05; ** *p* < 0.01; *** *p* < 0.001; **** *p* < 0.0001.

In order to evaluate the effect of factors secreted by different renal cell lines on the browning of hRAN explants, we measured changes in the expression of several browning markers. We observed that c/EBPβ LAP expression decreased due to the effect of NTc-CM and Tc-CM compared to Ctrl-CM (*** *p* < 0.001 and **** *p* < 0.0001, respectively; Figure 1a). In addition, HSL and PGC1α expression increased after incubation with Tc-CM compared to Ctrl-CM (** *p* < 0.01; Figure 1b,c). Also, PLIN 1 expression was higher in hRAN explants incubated with NTc-CM compared to explants treated with Tc-CM and Ctrl-CM (*** *p* < 0.001; Figure 1d). And UCP1 expression increased significantly in explants incubated with Tc-CM compared to NTc-CM (*** *p* < 0.001; Figure 1e). Surprisingly, UCP1 expression decreased due to the effect of NTc-CM vs. Ctrl-CM (* *p* < 0.05).

### 2.2. The Expression Profile of Adipocytokines and Their Receptors in hRAN Is Regulated by the CMs of Renal Epithelial Cell Lines

**Figure 2:**
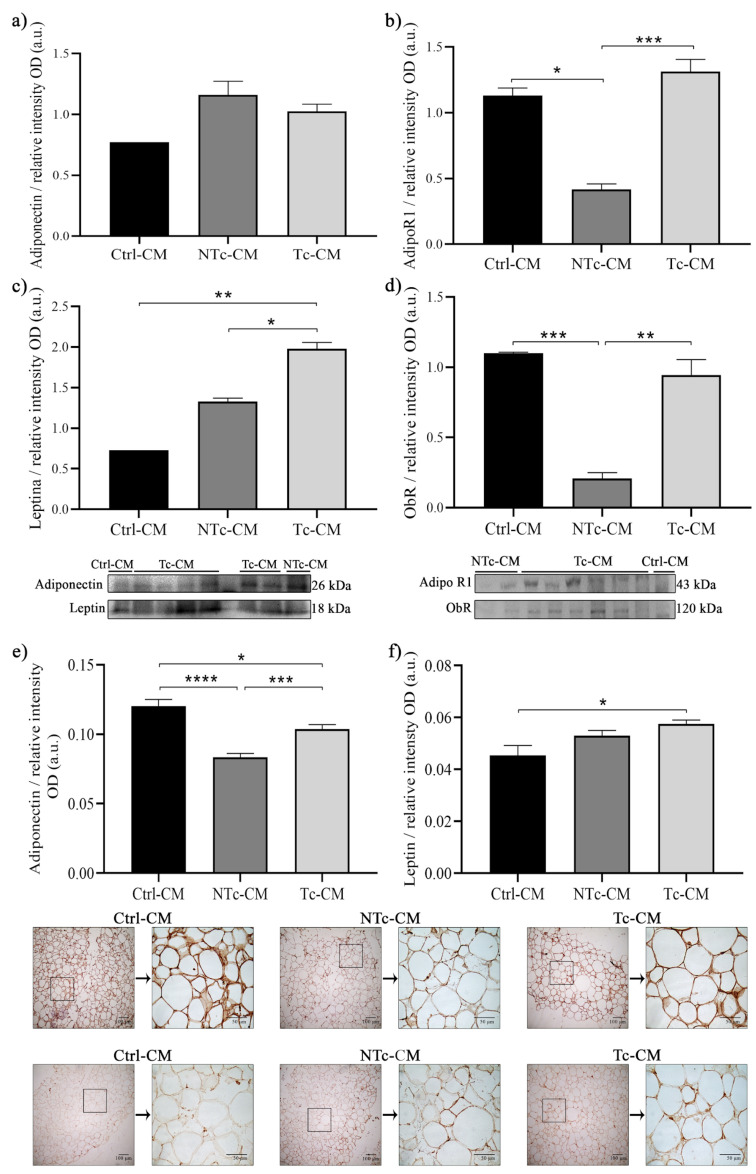
WB expression of: (**a**) adiponectin, (**b**) AdipoR1, (**c**) leptin, and (**d**) ObR and IHC expression of (**e**) adiponectin and (**f**) leptin in hRAN incubated with NTc- (*n* = 2), Tc- (*n* = 6), and Ctrl-CM (*n* = 2) for 48 h. The assay was performed in three technical replicates. Vertical bars represent the geometric mean of the data set, and horizontal bars represent SEM. Data was analyzed by Kruskal–Wallis test. The expression of each of the evaluated proteins was relative to the total amount of protein by Ponceau staining. The values of *n* refer to biological replicates. * *p* < 0.05; ** *p* < 0.01; *** *p* < 0.001 and **** *p* < 0.0001.

We evaluated changes in the expression of adiponectin and leptin, as well as their receptors, in hRAN incubated with the different CMs of the cell lines. By WB, we observed that the expression of adiponectin did not differ between treatments (Figure 2a). The tissue expression of AdipoR1 in hRAN decreased due to the effect of NTc-CM vs. Ctrl-CM (* *p* < 0.05). However, AdipoR1 increased in explants incubated with Tc-CM vs. NTc-CM (*** *p* < 0.001; Figure 2b). On the other hand, we observed an increase in leptin expression when the explants were incubated with Tc-CM vs. Ctrl-CM (** *p* < 0.01; Figure 2c). Finally, the expression of ObR in hRAN increased due to the effect of incubation with Tc-CM vs. NTc-CM (** *p* < 0.01) and decreased with NTc-CM vs. Ctrl-CM (*** *p* < 0.001; Figure 2d). Regarding IHC, we found that the expression of adiponectin decreased in hRAN treated with NTc-CM and Tc-CM vs. Ctrl-CM. In addition, Tc-CM increased the expression of adiponectin vs. NTc-CM (*** *p* < 0.001; Figure 2e). The leptin expression increased in hRAN after incubation with Tc-CM vs. Ctrl-CM (* *p* < 0.05; Figure 2f).

### 2.3. cT-CM and cNT-CM Increase the Metabolic Activity of hRAN

**Figure 3:**
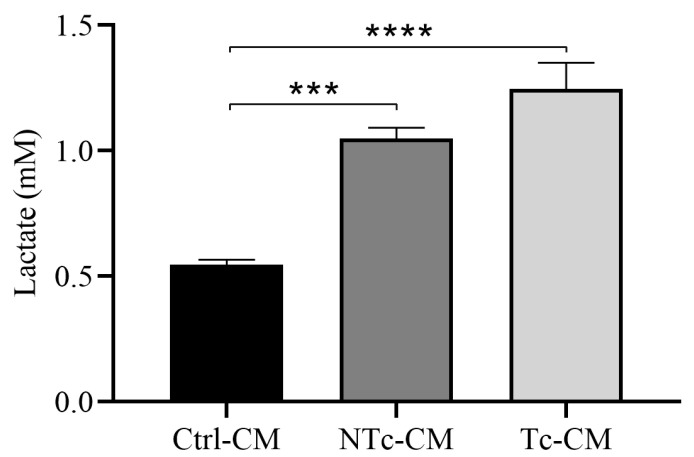
Lactate concentration in conditioned media from TAN explants incubated with NTc- (*n* = 2), Tc- (*n* = 6), and Ctrl-CM (*n* = 4) for 48 h. The assay was performed in three technical replicates. Vertical bars represent the geometric mean of the data set, and horizontal bars represent SEM. Data were analyzed using the Kruskal–Wallis test. The values of *n* refer to biological replicates. *** *p* < 0.001; **** *p* < 0.0001.

In order to evaluate changes in the metabolic activity of hRAN incubated with the different CMs, we measured the concentration of lactate produced by hRAN under the different experimental conditions. After treatment, we observed a significant increase in the lactate concentration in conditioned media from hRAN explants incubated with NTc-CM and Tc-CM vs. Ctrl-CM (*** *p* < 0.001 and **** *p* < 0.0001, respectively). We did not observe significant differences between NTc-CM and Tc-CM (Figure 3).

### 2.4. The Size of Adipocytes Incubated with Tc-CM Was Smaller than That of Adipocytes Incubated with NTc-CM

**Figure 4:**
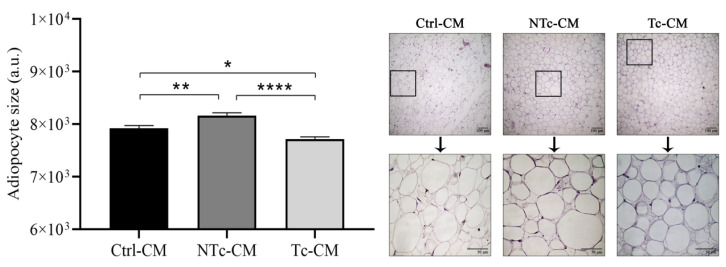
Size of hRAN adipocytes incubated with NTc- (*n* = 2), Tc- (*n* = 6), and Ctrl-CM (*n* = 4) for 48 h. In histological sections stained with HE, the area of adipocytes was measured using ImageJ software 1.54p. Vertical bars represent the geometric mean of the data set, and horizontal bars represent SEM. Quantification in the three tissue types was performed in 5 fields of each preparation. Data were analyzed using the Kruskal–Wallis test. The values of *n* refer to biological replicates. * *p* < 0.05; ** *p* < 0.01; **** *p* < 0.0001.

After incubating hRAN for 48 h with the different CMs, we observed a significant decrease in the size of adipocytes incubated with Tc-CM vs. NTc- and Ctrl-CM (**** *p* < 0.0001 and * *p* < 0.05, respectively; Figure 4). Furthermore, the size of hRAN adipocytes was smaller when incubated with MC-Ctrl vs. MC-NTc (** *p* < 0.01; Figure 4).

### 2.5. T-CM Stimulates hRAN Browning

**Figure 5:**
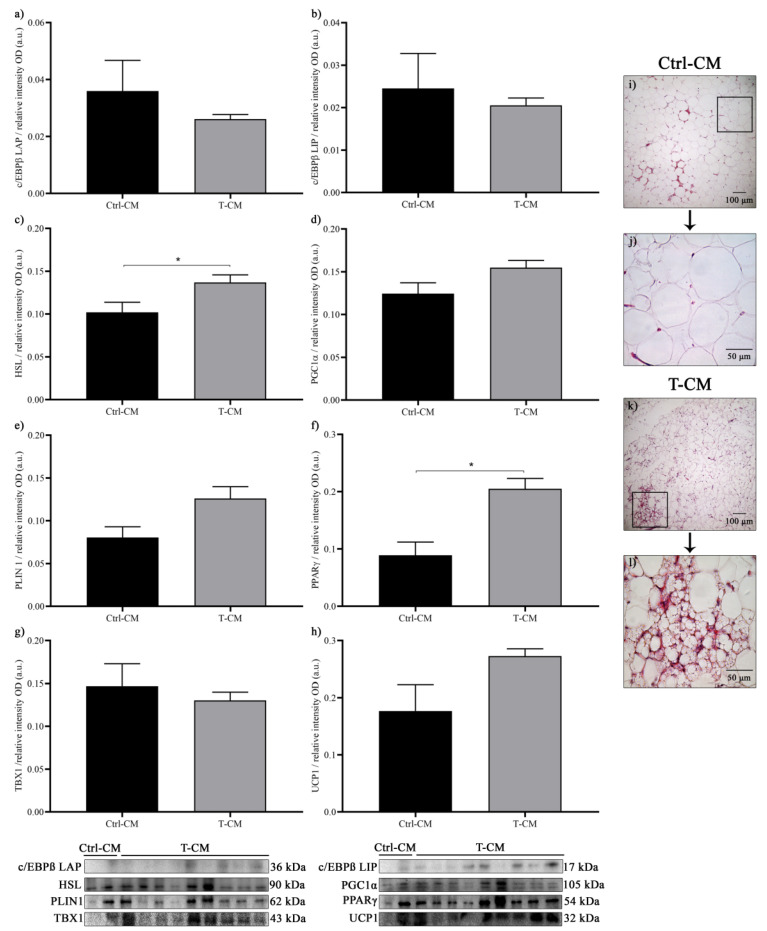
Protein expression, by WB, of (**a**) c/EBPß LAP (**b**) c/EBPß LIP, (**c**) HSL, (**d**) PGC1α, (**e**) PLIN, (**f**) PPARγ, (**g**) TBX1, and (**h**) UCP1 in hRAN explants incubated with T- (*n* = 9) and Ctrl-CM (*n* = 2) for 48 h. The assay was performed in three technical replicates. Vertical bars represent the geometric mean of the data set, and horizontal bars represent SEM. Data were analyzed using the Mann–Whitney or Welch tests, as appropriate. The expression of each of the evaluated proteins was relative to the total amount of protein by Ponceau staining. The values of *n* refer to biological replicates. * *p* < 0.05. (**i**–**l**) Histological sections stained with HE shows the histoarchitecture of hRAN explants incubated with T- and Ctrl-CM for 48 h. Photographs taken at 100× and 400×.

In order to confirm the results obtained with CMs from human renal tumor cell lines, we incubated hRAN fragments with CM from human renal tumor explants (T-CM) and evaluated changes in the expression of browning markers. After the treatment of hRAN with T- or Ctrl-CM, we observed no significant differences by WB in the expression of c/EBPß LAP, c/EBPß LIP, or TBX1 (Figure 5a,b,g), but we did observe a trend toward greater expression of PGC1α and UCP1 (Figure 5d,h). Regarding HSL and PPARγ, incubation of hRAN with T-CM resulted in increased expression of both proteins compared to Ctrl-CM (Figure 5c,f). When we evaluated histological sections of hRAN incubated with T-CM, we observed clusters of multilocular adipocytes typical of browned adipose tissue in HxE-stained sections (Figure 5i–l).

### 2.6. The Expression Profile of Adipocytokines in hRAN Is Regulated by T-CM

**Figure 6:**
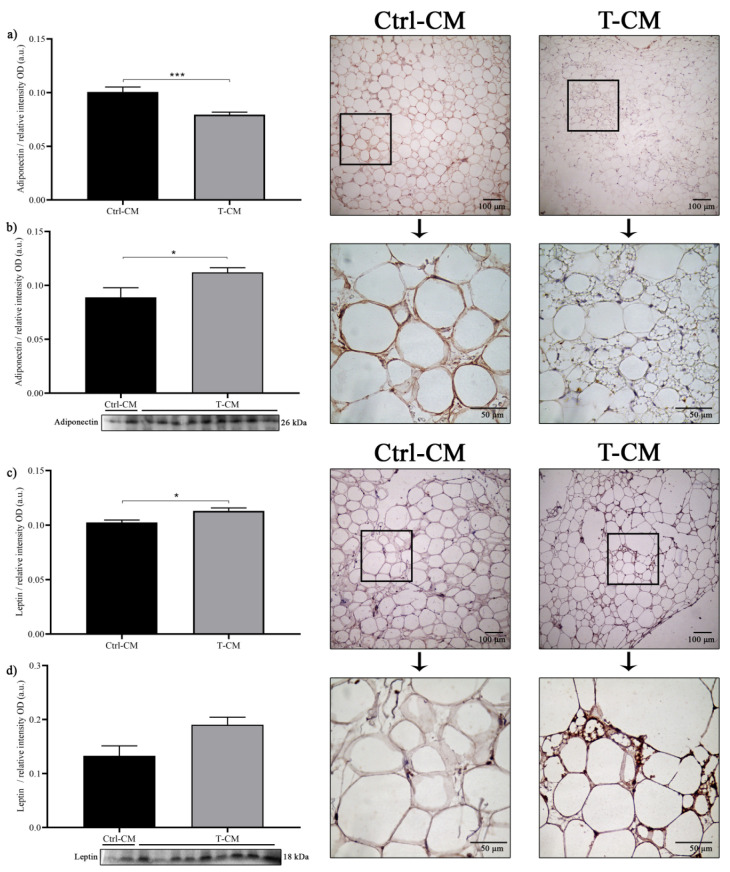
Protein expression of (**a**) adiponectin and (**c**) leptin by IHC and of (**b**) adiponectin and (**d**) Leptin by WB in hRAN explants incubated with T- (*n* = 9) and Ctrl-CM (*n* = 2) for 48 h. The assay was performed in three technical replicates. Vertical bars represent the geometric mean of the data set, and horizontal bars represent SEM. Data were analyzed using the Mann–Whitney test. The expression of each of the evaluated proteins was relative to the total amount of protein by Ponceau staining. The values of *n* refer to biological replicates. * *p* < 0.05; *** *p* < 0.001.

We evaluated the expression of adiponectin and leptin in hRAN explants incubated with T-CM and Ctrl-CM by IHC and WB. IHC showed a significant decrease regarding adiponectin expression in explants incubated with T-CM vs. Ctrl-CM (*** *p* < 0.001; Figure 6a). However, by WB, we found a significant increase in adiponectin expression in hRAN incubated with T-CM vs. MC-Ctrl (* *p* < 0.05; Figure 6b). In addition, we observed a significant increase in leptin expression in hRAN incubated with T-CM vs. Ctrl.CM by IHC (* *p* < 0.05; Figure 6c). This same tendency was observed using the WB technique (*p* = 0.06; Figure 6d).

### 2.7. T-CM Modifies the Expression of Proteins Involved in hRAN Metabolic Processes

**Figure 7:**
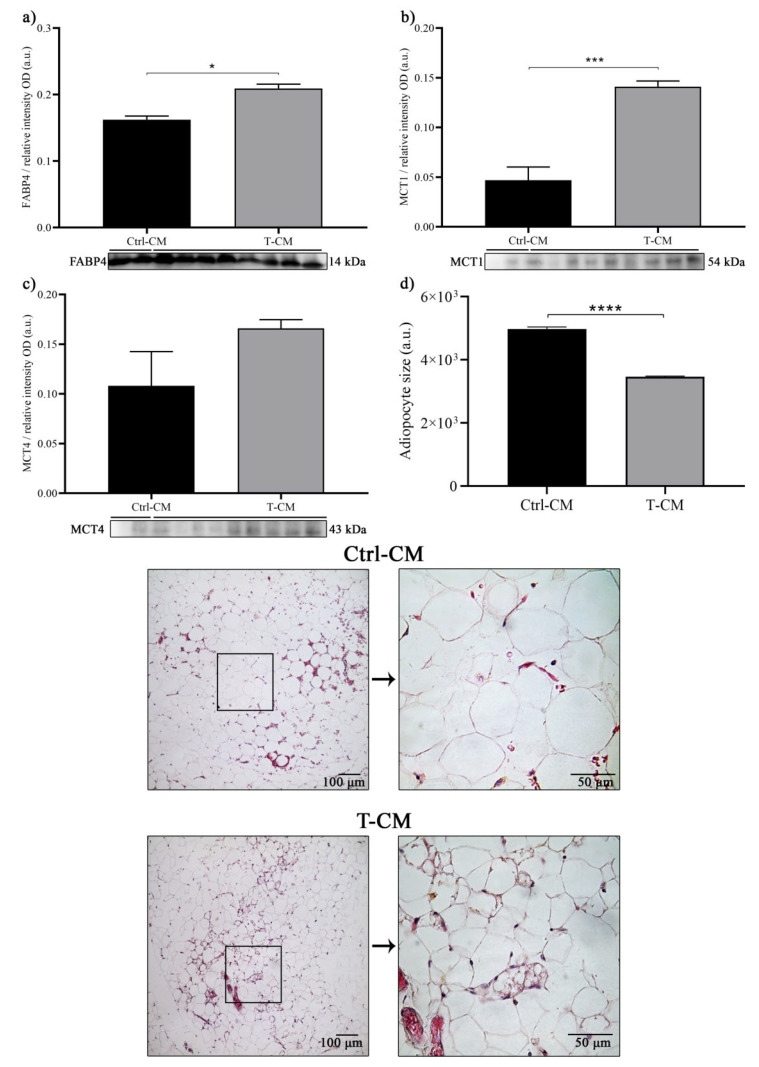
Expression, measured by WB, of (**a**) FABP4, (**b**) MCT1, and (**c**) MCT4 in hRAN fragments incubated with T- (*n* = 9) and Ctrl-CM (*n* = 2) for 48 h. The assay was performed in three technical replicates. The expression of each of the evaluated proteins was relative to the total amount of protein by Ponceau staining. (**d**) Size of hRAN adipocytes incubated with T- and Ctrl-CM for 48 h. Adipocyte area was measured in HE-stained histological sections using ImageJ software. Vertical bars represent the geometric mean of the data set, and horizontal bars represent SEM. Data were analyzed by the Mann–Whitney test. The values of *n* refer to biological replicates. * *p* < 0.05, *** *p* < 0.001, **** *p* < 0.0001.

In order to evaluate the possible metabolic reprogramming of hRAN by soluble factors secreted by the renal tumor, we evaluated changes in the expression of proteins involved in tissue metabolism and in its histoarchitecture. We observed a significant increase in the expression of FABP4 (* *p* < 0.05) and MCT1 (*** *p* < 0.001) in hRAN incubated with T-CM compared with Ctrl-CM (Figure 7a,b). However, we did not observe significant differences between treatments for MCT4 (Figure 7c). In addition, we observed that hRAN incubated with T-CM exhibited smaller adipocytes than hRAN incubated with Ctrl-CM (**** *p* < 0.0001; Figure 7d).

### 2.8. T-CM Modifies the Metabolic Activity of hRAN

**Figure 8:**
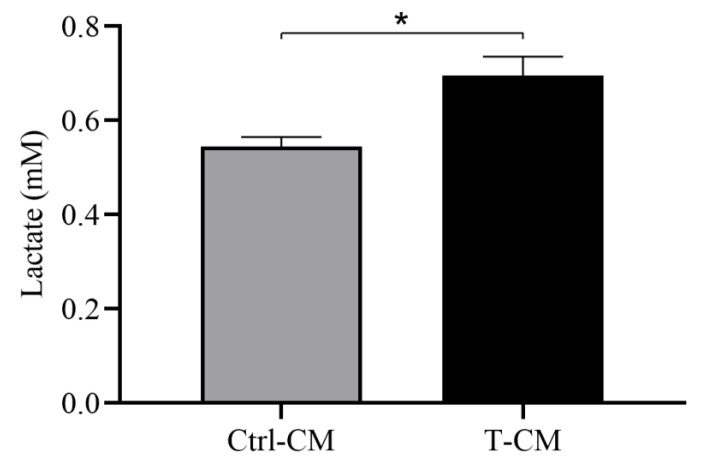
Lactate concentration (mM) in hRAN CMs incubated with T- (*n* = 9) and Ctrl-CM (*n* = 3) for 48 h. The assay was performed in three technical replicates. Vertical bars represent the geometric mean of the data set, and horizontal bars represent SEM. Data were analyzed using the Mann–Whitney test. The values of *n* refer to biological replicates. * *p* < 0.05.

To continue evaluating the metabolic activity of adipose tissue explants incubated with the different CMs, we measured the concentration of lactate produced by hRAN after being incubated for 48 h with T- or Ctrl-CM. We observed an increase in the lactate concentration in the CM for hRAN explants incubated with T-CM vs. Ctrl-CM (* *p* < 0.05; Figure 8).

### 2.9. T-CM Modulates the Expression of Steroid Hormone Receptors in hRAN

**Figure 9:**
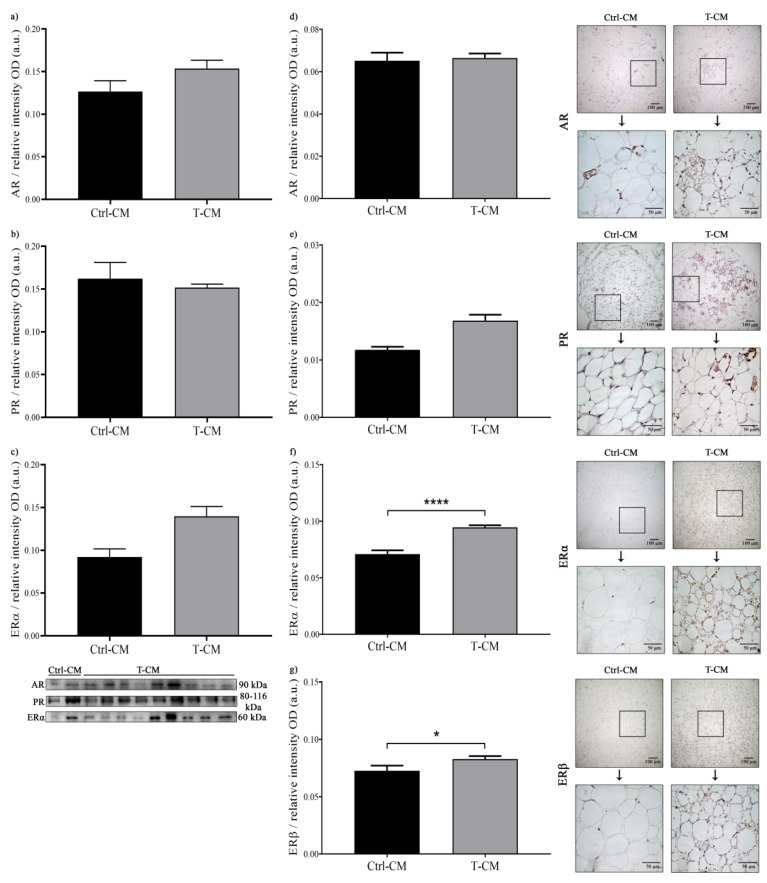
Expression of (**a**) AR, (**b**) PR, and (**c**) ERα measured by WB and (**d**) AR, (**e**) PR, (**f**) Erα, and (**g**) ERβ measured by IHC in hRAN explants treated with T- (*n* = 9) and Ctrl-CM (*n* = 2) for 48 h. The assay was performed in three technical replicates. The expression of each of the evaluated proteins was relative to the total amount of protein by Ponceau staining. Vertical bars represent the geometric mean of the data set, and horizontal bars represent SEM. Data were analyzed using Student’s *t* test or Mann–Whitney test, as appropriate. The values of *n* refer to biological replicates. * *p* < 0.05, **** *p* < 0.0001.

Since estrogens, androgens, and progesterone modulate proliferation, lipolytic activity, and adipocytokine production in AT, and since the dysregulation of these steroid hormones and their receptors can promote cancer development, we wanted to evaluate whether the renal tumor was able to modulate the response of the surrounding AT to these hormones. After 48 h of incubation with T-CM and Ctrl-CM, we did not observe significant differences in the expression, by WB, of AR and PR receptors in hRAN (Figure 9a,b), but there was a tendency for ERα to increase in hRAN incubated with T-CM (Figure 9c). Regarding IHC, we also did not observe differences in the expression of AR and PR (Figure 9d,e), but we observed a significant increase in the expression of ERα (**** *p* < 0.0001) and ERβ (* *p* < 0.05) (Figure 9f,g).

## 3. Discussion

Based on previous results from our group [17,18,33], where we characterized hRAN and hRAT fragments and evaluated the effect of CMs from these explants on renal cells, we decided to study the reverse dialogue. Therefore, we incubated hRAN fragments with CMs from tumors (T-CM) or tumor and nontumor epithelial renal cell lines (Tc-CM or NTc-CM, respectively) and evaluated the changes that this incubation produced in the hRAN fragments. Thus, we observed that hRAN incubated with Tc-CM increases the expression of HSL, PGC1α, and UCP1 (Figure 1b,c,e) and decreases the expression of PLIN1 (Figure 1d) compared to NTc-CM. In line with these results, we observed an increase in HSL and also PPARγ after hRAN treatment with T-CM vs. Ctrl-CM (Figure 5c,f). We also observed that in hRAN treated with Tc-CM or T-CM the adipocytes were smaller than the adipocytes of hRAN incubated with control medium (Figure 4 and Figure 7d, respectively).

The adipocytes present in the tumor microenvironment exhibit an altered phenotype and are called cancer-associated adipocytes (CAAs). Our results show significant changes in the expression of differentiation and browning markers of hRAN incubated with Tc-CM and T-CM. Grigoras & Amalinei 2023 [34] reported that CAAs from patients with colorectal cancer are smaller in size and have elevated HSL expression compared to mature adipocytes. The increased expression of HSL, decreased PLIN, and changes in adipocyte size suggest that hRAN undergoes lipolysis triggered by factors released by tumor cells. Other authors have demonstrated that adipocytes co-cultured with prostate and breast cancer cell lines undergo delipidation [10,35]. On the other hand, although we did not observe significant differences in the expression of specific browning markers such as UCP1 in hRAN incubated with T-CM, we did observe an increase in PPARγ expression after treatment of the explants. PPARγ is a regulator of adipocyte dedifferentiation and the phenotypic acquisition of CAAs and also regulates lipid and glucose metabolism [34]. Likewise, we detected the presence of multilocular adipocyte islands in hRAN treated with T-CM (Figure 5).

Regarding adiponectin expression, the results were mixed. In hRAN treated with CM obtained from cell lines (NTc- or Tc-CM), we did not observe significant differences between treatments, at least by WB, but we did observe an increase in the expression of the adiponectin receptor AdipoR1 in hRAN treated with Tc-CM vs. NTc-CM. Surprisingly, we observed a significant decrease in AdipoR1 expression in hRAN incubated with NTc-CM vs. Ctrl-CM (Figure 2b). Nevertheless, by IHC, we did observe a significant increase in hRAN explants incubated with Tc-CM vs. NTc-CM; also, we observed a significant decrease in adiponectin expression in Tc-CM vs. Ctrl-CM (Figure 2e). Regarding adiponectin expression in hRAN explants treated with T-CM, we observed a significant decrease vs. Ctrl-CM, by IHC. However, by WB, we observed the opposite; that is, adiponectin expression increased in hRAN incubated with T-CM vs. Ctrl-CM (Figure 6a,b). The observed discrepancy between protein quantification methods—where adiponectin levels were elevated in WB lysates but diminished in IHC staining within the peritumoral AT—suggests a tumor-induced alteration in adiponectin dynamics [36]. We hypothesize that the ccRCC microenvironment triggers an accelerated secretion rate of adiponectin from perirenal adipocytes [15]. While WB captures the total protein pool, including the enriched interstitial fraction, IHC predominantly reflects the depleted intracellular storage [37]. Regarding leptin, the results obtained with the different methodological approaches were congruent. We observed an increase in leptin expression in hRAN incubated with Tc-CM (Figure 2c,f) and T-CM (Figure 6c,d) vs. NTc-CM and Ctrl-CM, respectively. In addition, we observed an increase in the expression of the leptin receptor ObR in hRAN incubated with Tc-CM vs. NTc-CM (Figure 2d). These results are consistent with previous findings, in which increased leptin and ObR levels were observed in AT explants from RCC patients compared to AT from healthy donors [17,33]. There is consensus on the lipolytic role of leptin [38,39,40] and antilipolytic role of adiponectin [41,42]. These results support the notion that adipocytes undergo lipolysis.

The sustained proliferation and survival of tumor cells in neoplastic diseases entails a high energy demand, constant biosynthetic requirements, and the maintenance of redox balance. To meet all these requirements, tumor cells undergo a metabolic reprogramming process, which is currently considered a hallmark of cancer [1,43]. In addition to aerobic glycolysis and the Krebs cycle, tumor cells explore other pathways that, for nontumor cells, are unconventional in terms of energy yield. One of these is lactic acid fermentation, a process by which pyruvate molecules formed after the breakdown of glucose are converted into lactate. This mechanism typically occurs under anaerobic conditions, but it has been described that tumor cells, even under aerobic conditions, choose this metabolic pathway. This phenomenon, known as the Warburg effect (WE), allows tumor cells to survive in hostile environments and resist cell death signals [2,7]. In this reprogramming process, altered lipid metabolism is a crucial event that occurs in several types of cancer [44]. In turn, tumor cells obtain exogenous lipids. These lipids are used for membrane synthesis during cell growth, act as substrates for ATP synthesis, and participate in signaling pathways involved in cell proliferation and survival [45]. In addition to the metabolic reprogramming of tumor cells, the tumor microenvironment also undergoes a process of metabolic rearrangement. To assess whether renal tumors are truly capable of regulating the metabolic rearrangement of the surrounding adipose tissue, we studied changes in the expression of FABP4, MCT1, and MCT4 in hRAN fragments incubated with the different CMs. FABP4 is a key indicator of adipose dysfunction, as it coordinates lipid trafficking and metabolic signaling between adipocytes and the tumor microenvironment. Evaluating their levels of expression is crucial for assessing the metabolic reprogramming of peritumoral fat and its contribution to cancer-associated lipid mobilization [46]. Furthermore, lactate uptake by cells occurs via the high-affinity transporter MCT1, while lactate derived from glycolysis is released via the low-affinity transporter MCT4 [47]. Thus, we observed a significant increase in the expression of FABP4 and MCT1 in explants treated with T-CM vs. Ctrl-CM (Figure 7a,b). We also evaluated lactate secretion in CMs obtained from hRAN previously incubated with the different CMs (cells and tumor). We observed that both hRAN treated with Tc-CM (Figure 3) and that treated with T-CM (Figure 8) secreted a greater amount of lactate than hRAN incubated with control medium. Strikingly, hRAN incubated with NTc-CM released greater amounts of lactate than that incubated with Ctrl-CM (Figure 3). Regarding FABP4 expression, the literature reports disparate results: Pagnotta et al. (2023) [48] reported a decrease in FABP4 expression in preadipocytes incubated with AT-CM, while Wei et al. (2023) [49] reported no differences in the expression of this transporter in CAAs from patients with RCC. This difference could be due to the methodological approaches employed by the authors. In the first case, adipocyte precursor cells were incubated with CM from healthy mammary adipose tissue and peritumoral mammary adipose tissue, and changes in expression were observed after 72 h of treatment, while our treatment time was 48 h. In the second case, FABP4 expression in tumoral perirenal AT explants and healthy perirenal AT explants was compared.

In addition to regulating energy balance, adipose tissue is the largest extragonadal organ producing steroid hormones due to its aromatase activity [23,50]. Given the regulatory role of sex hormones, we decided to evaluate the status of estrogen, androgen, and progesterone receptors in hRAN fragments treated with T-CM. After the incubation of hRAN with the different CMs, we found no significant differences in the expression of the androgen receptor (AR), progesterone receptor (PR), and estrogen receptor α (ERα), although for the latter, we observed an upward tendency in hRAN incubated with T-CM vs. Ctrl-CM (Figure 9a,c, WB technique). When evaluating receptor expression by IHC, we also did not observe significant differences in AR expression (Figure 9d), but there was a tendency for PR to increase in hRAN incubated with T-CM vs. Ctrl-CM (Figure 9e). Regarding estrogen receptors, we observed a significant increase in the expression of both ERα and ERβ (Figure 9f,g). Sex hormones participate in adipocyte proliferation and differentiation, lipid consumption, lipase activity, and adipocytokine synthesis [31]. It has been reported that ERα in adipocytes plays an important role in maintaining AT function and protecting them from inflammatory damage, due to its lipolysis-stimulating effect [51]. We have observed, in histological sections, that the largest foci of positive labeling for steroid hormone receptors coincide with areas of multilocular adipocytes (Figure 9). Estrogen receptors could stimulate the browning of AT by stimulating mitochondriogenesis and the expression of UCP1 [52,53]. Furthermore, in vitro studies suggest that leptin synthesis and secretion are controlled by sex steroids; the interaction of estrogen with ERα in 3T3-L1 adipocytes induces leptin expression, while binding with ERβ exerts the opposite effect [53,54]. Thus, there could be a link between changes in sex hormone receptors, lipolysis levels, and the browning of AT regulated by factors secreted by renal tumors.

In conclusion, due to the metabolic reprogramming that renal tumors undergo, adipose tissue is exposed to a series of changes to overcome the selection pressure that results from the development of a tumor mass. The new metabolic pathways explored by the tumor, such as fatty acid oxidation (FAO) and aerobic glycolysis, generate a loss of lipid content in AT, microenvironmental acidosis, and metabolic pseudohypoxia [34,47]. The dedifferentiation and browning of hRAN, as well as the change in the adipocytokine expression profile and the increase in lactatogenesis, are consequences of the presence of the tumor. These results support the hypothesis that ccRCC induces dedifferentiation and browning of peritumoral fat by transforming hRAN into a metabolically “activated” tissue that drives tumor progression through fatty acid mobilization and aerobic glycolysis [6,21,22].

Some of the authors mentioned previously demonstrate bidirectional communication between tumor cell lines and adipocyte precursors or evaluate the status of AT explants from healthy patients or RCC patients [44,45]. However, this study, together with previous results already published by our group, provides the first evidence of a bidirectional interaction between human adipose tissue explants and ccRCC tumors using an ex vivo model. While our results are conclusive regarding the changes that adipose tissue surrounding a renal tumor undergoes in response to the tumor’s presence, this is a largely descriptive study. The perirenal adipose tissue explants used in this work were obtained from two distinct donors and subsequently fragmented, with each piece being treated with a different conditioned medium (Tc-CM, NTc-CM, T-CM, Ctrl-CM). However, the reliance on *n* = 2 hRAN donors represents a limitation of this work, as it may potentially restrict the generalizability of the observed findings to a more diverse patient population. Future experiments will seek to elucidate the cellular and molecular mechanisms involved in the effects described here.

## 4. Materials and Methods

### 4.1. Reagents

Reagents were from Sigma Chemical Co. (St. Louis, MO, USA); tissue culture flasks, dishes, and multi-well plates were from Falcon Orange Scientific (Graignette Business Park, Braine-l’Alleud, Belgium); and culture media for both tissue and cell lines and supplements were from Gibco BRL (Carlsbad, CA, USA).

### 4.2. Sample Collection and Handling

Patients with suspected kidney cancer (*n* = 55) and healthy kidney donors (*n* = 20) were enrolled. After signing the informed consent form, subjects were interviewed using a standard questionnaire that requested information about socio-demographic, medical, and lifestyle factors. Human ccRCC tumor explants (*n* = 12) were obtained from ccRCC live patients who had not received previous chemotherapy or radiotherapy treatment. For this particular study, in which we used several fragments of adipose tissue, human adipose tissue explants from normal kidney (hRAN, *n* = 2) were obtained from live kidney donors; the adipose tissue fragment was collected 1 cm away from the kidney’s middle zone (middle pole). In all cases, biopsies were taken distant from the adrenal gland (source of norepinephrine). The median body mass index (BMI) of patients was 24.9 kg/m^2^. The BMI (kg/m^2^) was calculated as weight (kg) divided by height (m) squared. Samples were transported in PBS 1X and processed immediately. On average, 2 h elapsed from the acquisition of the surgical sample until it was processed under a sterile laminar flow hood. The project was approved by the Medical School’s ethics committee (Universidad Nacional de Cuyo, Argentina) according to the Declaration of Helsinki on experimentation with human subjects. All patients gave their informed consent to undergo tissue harvesting for this research [19].

### 4.3. Preparation of Conditioned Medium from Renal Tumors (T-CM)

Human renal tumor explants were processed in laminar flow cabinets. Tissues were placed in sterile T25 cell culture flasks (Jet BIOFIL^®^ CellATTACH^®^, Guangzhou, China) with D-MEM/F12 medium (Invitrogen, ThermoFisher Scientific, Waltham, MA, USA) (1 mL per g of tissue) without FBS and incubated at 37 °C in a Invitrogen, ThermoFisher Scientific (Waltham, MA, USA) culture oven with 5% CO_2_. After 1 h, the medium was replaced with fresh medium, and the explants were incubated for 24 h. After that time, the conditioned medium was collected, filtered with 0.22 μm Ministar^®^ Syringe Filters (Sartorius, Göttingen, Germany), aliquoted, and stored at −80 °C until use.

### 4.4. Preparation of Conditioned Media from Nontumor (NTc-CM) and Tumoral (Tc-CM) Human Renal Epithelial Cell Lines

Medium conditioning was done in sterile Petri dishes (Jet BIOFIL^®^ CellATTACH^®^, China). For this purpose, 5 × 10^5^ cells were seeded per plate, grown in the corresponding medium (D-MEM/F12 for HK-2 or RPMI 1640 for 786-O, ACHN, and Caki-1) at 10% FBS (Internegocios, Mercedes, Argentina) and incubated at 37 °C with 5% CO_2_. Once 90% confluence was reached, the medium was discarded, the plates were washed with sterile PBS 1X, and the culture medium corresponding to the different lines was added at 2% FBS. After one hour, the medium was discarded, and fresh medium was added. After 24 h, the medium was collected, filtered with 0.22 μm Ministar^®^ Syringe Filters (Sartorius, Germany), aliquoted into Eppendorf tubes (Eppendorf, Enfield, CT, USA), and stored at −80 °C until use.

### 4.5. Treatment of hRAN with NTc-CM, Tc-CM, and T-CM

hRAN explants, 10–12 fragments of approximately 1 g each, were obtained from multiple patients. The AT fragments were incubated in 12-well plates (Jet BIOFIL^®^ CellATTACH^®^, China) and treated with the conditioned media ([cell line CM: 50% CM from cell lines + 50% M199], [tumor CM: 50% CM + 1% FBS + 50% M199], [control CM: 50% D-MEM/F12 + 2% FBS + 50% M199]). All assays were performed in duplicate (2 fragments per treatment) at a final volume of 1 mL/well. The explants were incubated at 37 °C in a 5% CO_2_ culture chamber for 48 h, with the treatments replaced after 24 h. Of the two explants per treatment, one was fixed in 4% paraformaldehyde for subsequent histological processing, and the other was frozen at −80 °C for molecular biology assays.

### 4.6. H&E Staining

Tissues (hRAT and hRAN) were fixed in 4% paraformaldehyde and embedded in paraffin. They were then cut into sections of 5 μm thickness with a microtome, deparaffinized, and stained with hematoxylin–eosin (H&E). Five images per field were taken with a Nikon Eclipse E200 Microscope fitted with a digital still camera, Micrometric SE Premium (Nikon Corp., Tokyo, Japan), at 100× magnification [18].

### 4.7. Immunohistochemistry

Serial 10 µm cuts were performed on the same tissue samples embedded in paraffin used for H&E staining. Briefly, hRAN slides were first deparaffinized, and then heat-mediated antigen retrieval, endogenous peroxidase blocking, and nonspecific tissue blocking were performed. Slides were then incubated with the different primary antibodies at 4 °C: adiponectin (mouse monoclonal, ab22554, dilution 1:200), AdipoR1 (rabbit monoclonal, ab126611, 1:50), AR (rabbit monoclonal, ab108341, dilution 1:50), ERα (rabbit monoclonal, ab32063, dilution 1:50), ERβ (mouse monoclonal, ab187291, dilution 1:500), leptin (rabbit polyclonal, ab117751, dilution 1:100), ObR (mouse monoclonal, sc8391, dilution 1:50), and PR (rabbit polyclonal, sc539, dilution 1:100). After that, slides were incubated with an anti-rabbit biotinylated secondary IgG antibody. Finally, slides were incubated with peroxidase-conjugated streptavidin. Peroxidase reaction was performed with chromogen 3,3′-diaminobenzidine (DAB) (DAKO LSAB+Kit, HRP). Hematoxylin counterstaining was performed. Serial cuts incubated in the absence of the primary antibody were used as negative controls. Images were taken with a Nikon Eclipse E200 Microscope fitted with a Micrometric SE Premium (Nikon Corp., Japan) digital still camera at 10× and 40× magnification. DAB staining quantification in the three tissue types was performed in 5 fields of each slide [18].

### 4.8. Lysis of Human hRAN

To extract proteins from the TA samples, 200 mg of each tissue was weighed and placed in 1 mL of Ripa buffer with protease inhibitor (Complete Tablets Easy Pack, Roche 04693116001, Germany) at a dilution of 1:100. The samples were homogenized in an Ultra-Turrax (Janke and Kunkel, IKA-WERK, Higashiosaka, Japan) in the cold. Once the samples were homogenized, they were centrifuged at 12,000 rpm for 15 min at 4 °C. The supernatant was recovered without dragging the fatty residue and stored at −20 °C. Total proteins were quantified by the Micro BCA Pierce method, according to the manufacturer’s instructions (Thermo Scientific Pierce BCA Protein Assay Kit; Waltham, MA, USA).

### 4.9. Western Blot Analysis

In order to evaluate protein expression levels, WB was performed. Adiponectin, AR, c/EBPβ LAP, ERα, FABP4, HSL, MCT1, MCT4, PCG1α, PLIN 1, PPARγ, PR, TBX1, and UPC1 were measured after incubation of hRAN with the different CMs obtained. Proteins were separated on an SDS-PAGE 12% acrylamide gel and electrotransferred to a PVDF membrane (Bio-Rad, Hercules, CA, USA). The membrane was later blocked with human 10% serum albumin (0055 K Sigma-Aldrich, Merck Group Co. St. Louis, MO, USA) and then incubated with the different antibodies ON at 4 °C: adiponectin (mouse monoclonal, ab22554, dilution 1:500), AdipoR1 (rabbit monoclonal, ab126611, 1:1000), AR (rabbit monoclonal, ab108341, 1:200), c/EBPβ (rabbit monoclonal, ab32358, dilution 1:1000), ERα (rabbit monoclonal, ab32063, dilution 1:1000), ERβ (mouse monoclonal, ab187291, dilution 1:2000), FABP4 (rabbit monoclonal, ab92501, dilution 1:5000), HSL (rabbit polyclonal, ab45422, dilution 1:1000), leptin (rabbit polyclonal, ab117751, dilution 1:1000), MCT1 (rabbit monoclonal, ab90582, dilution 1:1000), MCT4 (rabbit polyclonal, sc50329, dilution 1:500), ObR (mouse monoclonal, sc8391, dilution 1:500), PLIN1 (rabbit polyclonal, ab3526, dilution 1:2000), PGC1α (rabbit polyclonal, ab54481, dilution 1:1000), PPARγ (mouse monoclonal, sc7273, dilution 1:500), PR (rabbit polyclonal, sc539, dilution 1:500), TBX1 (rabbit polyclonal, u6382, dilution 1:500), and UCP1 (rabbit polyclonal, sc539, dilution 1:1000). After that, the membranes were washed and incubated with proper secondary antibodies conjugated with biotin, and subsequently, the signal was amplified with streptoavidin–peroxidase conjugation 1:5000 (Calbiochem, Merk, Saint Louis, MO, USA). Antibody complexes were visualized by means of chemiluminescence (ECL; GE Helathcare, Chicago, IL, USA). Membrane-exposed images were obtained with the Chemidoc MP system (Bio-Rad, USA), and bands were quantified by densitometry using the FIJI Image processing package 1.54p (NIH, Bethesda, MD, USA) [18]. The expression of each of the evaluated proteins was relative to the total amount of protein by Ponceau staining [55]. The Ponceau-stained membranes that allowed quantification of the total proteins corresponding to Figures S1 and S2 are shown in Supplementary Figure S1; the membranes corresponding to Figure 5, Figure 6, Figure 7 and Figure 9 are shown in Supplementary Figure S2.

### 4.10. Lactate Determination in CMs

A lactate determination kit (Wiener Lab, REF: 1999795, Rosario, Argentina) was used, according to the manufacturer’s instructions. In 96-well plates (Jet BIOFIL^®^ CellATTACH^®^, China), 165 μL of reagent A was added per well, along with 2 μL of the sample to be measured and 33 μL of reagent B. The mixing of the different components was carried out under refrigeration to prevent possible enzymatic reactions. The plate was then incubated at room temperature for 1 min and then at 37 °C for 15 min. Finally, the absorbance at 570 nm was measured using a plate reader (MULTISKAN EX Thermo Fisher Scientific, Waltham, MA, USA).

## 5. Conclusions

Due to the metabolic reprogramming that renal tumors undergo, adipose tissue is exposed to a series of changes to overcome the selection pressure that results from the development of a tumor mass. The dedifferentiation and browning of hRAN, as well as the change in the adipocytokine expression profile and the increase in lactatogenesis, are consequences of the presence of the tumor. Taking account of our previous works, this study provides evidence of bidirectional communications between human adipose tissue and ccRCC tumors.

## Data Availability

The data sets generated and analyzed during this study are already publicly available in the CONICET Data Repository at the following link: https://ri.conicet.gov.ar/handle/11336/276412 (accesed on 2 January 2026).

## Supplementary Materials

The following supporting information can be downloaded at: https://www.mdpi.com/article/10.3390/ijms27031528/s1.
