# Supplementary material for: Adipose Tissue and Renal Carcinoma: A Protumor Metabolic and Endocrine Alliance
Source: Int J Mol Sci. 2026 Feb 4;27(3):1528. doi: 10.3390/ijms27031528 (PMC12898272; doi:10.3390/ijms27031528)
Supplement: Supplementary file 1 [file ijms-27-01528-s001.zip › Supplementary figure S1.pdf]

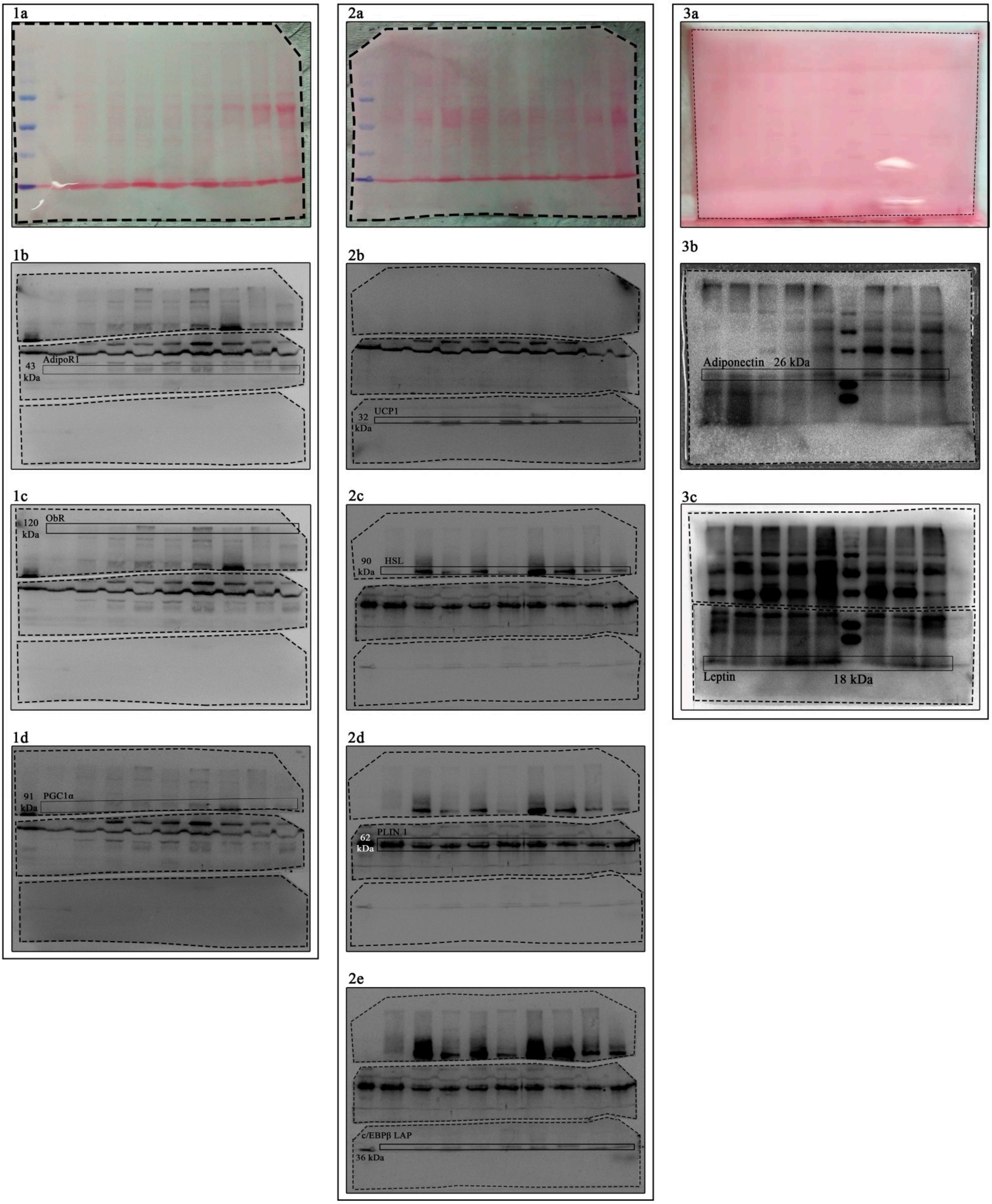

Supplementary figure S1: Full uncropped gels and blots of TAN explant lysis incubated with CMs of human renal epithelial cell lines.

1a: Pounceau; 1b: AdipoR; 1c: ObR; 1d: PGC1α

2a: Pounceau; 2b: UCP1; 2c: HSL, 2d: PLIN 1; 2e: c/EBPβ LIP

3a: Pounceau; 3b: Adiponectin; 3c: Leptin
