# Supplementary material for: Adipose Tissue and Renal Carcinoma: A Protumor Metabolic and Endocrine Alliance
Source: Int J Mol Sci. 2026 Feb 4;27(3):1528. doi: 10.3390/ijms27031528 (PMC12898272; doi:10.3390/ijms27031528)
Supplement: Supplementary file 1 [file ijms-27-01528-s001.zip › Supplementary figure S2.pdf]

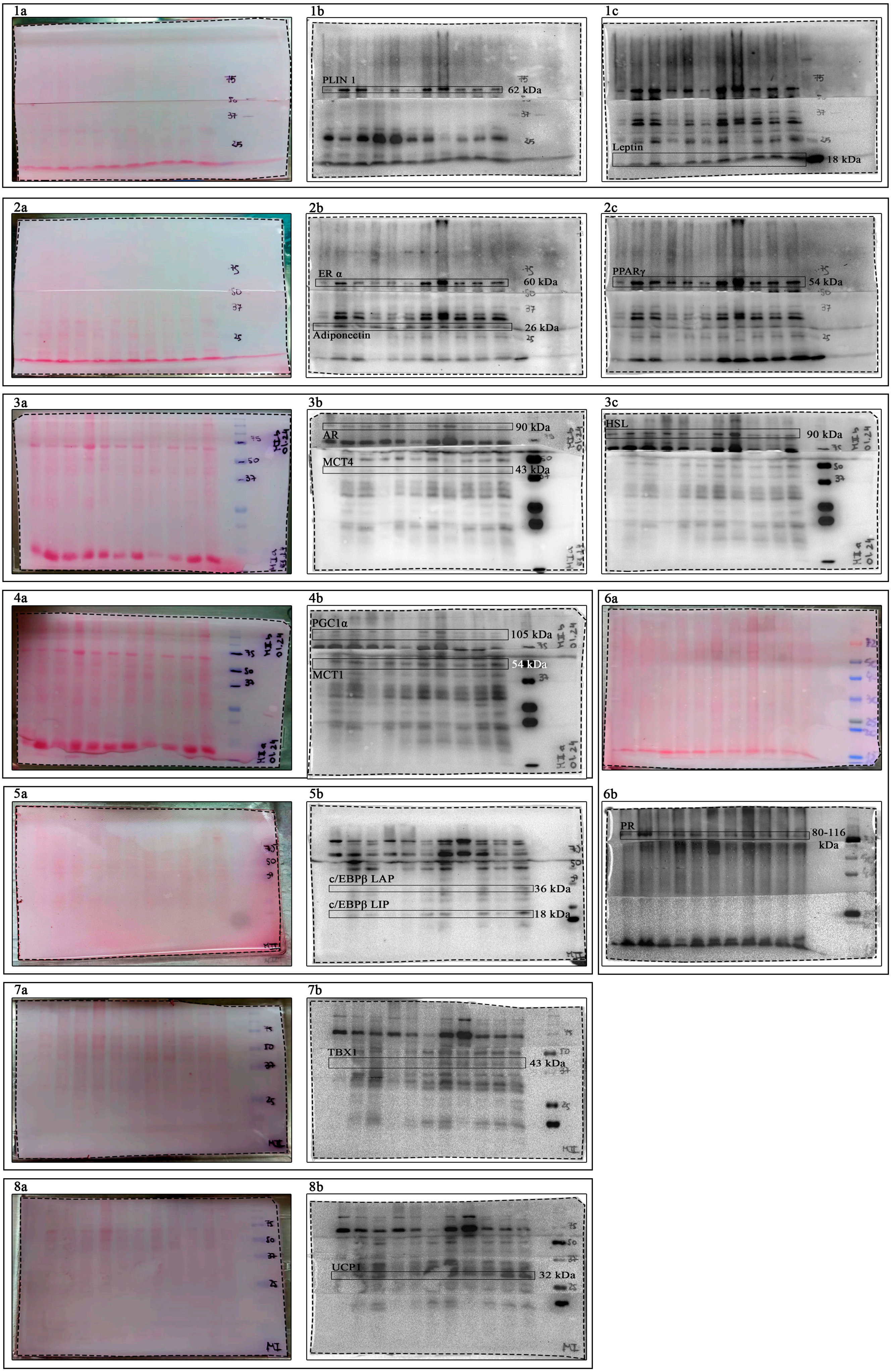
1a
1b
PLIN 1
62 kDa
1c
Leptin
18 kDa
2a
2b
ER α
60 kDa
Adiponectin
26 kDa
2c
PPARγ
54 kDa
3a
3b
90 kDa
AR
MCT4
43 kDa
3c
HSL
90 kDa
4a
4b
PGC1α
105 kDa
54 kDa
MCT1
6a
5a
5b
c/EBPβ LAP
36 kDa
c/EBPβ LIP
18 kDa
6b
PR
80-116
kDa
7a
7b
TBX1
43 kDa
8a
8b
UCP1
32 kDa

Supplementary figure S2: Full uncropped gels and blots of TAN lysis explants incubated with MC from human renal tumors.

1a: Pounceau; 1b: PLIN 1; 1c: Leptin

2a: Pounceau; 2b: ERα and Adiponectin; 2c: PPARɣ

3a: Pounceau; 3b: AR and MCT4, 3c: HSL

4a: Pounceau; 4b: PGC1α and MCT1

5a: Pounceau; 5b: c/EBPβ LAP and c/EBPβ LIP

6a: Pounceau; 6b: PR

7a: Pounceau, 7b: TBX1

8a: Pounceau, 8b: UCP1
